# Effect of ageing on the physicochemical properties of human faeces in the context of onsite sanitation

**DOI:** 10.1016/j.envc.2023.100717

**Published:** 2023-04

**Authors:** T.M. Chatema, E. Mercer, S. Septien, J. Pocock, C.A. Buckley

**Affiliations:** a WASH R&D Centre (formerly the Pollution Research Group), University of KwaZulu-Natal, Howard College, 4041, Durban, South Africa; b Chemical Engineering, University of KwaZulu-Natal, Howard College, 4041, Durban, South Africa

**Keywords:** Source separation sanitation, Drying, Moisture boundness, Rheology, Nutrient analysis, Thermal properties

## Abstract

Sanitation systems involving onsite storage of faecal matter cause excreta to undergo transformation and primary treatment in-situ. However, little is known about the transformation pathway followed by fresh faeces while contained in situ. The current paper investigated this transformation under ambient conditions during a 16-week in-situ-storage period. Moisture content, drying kinetics, rheological, physicochemical, and thermal properties were analysed to determine the effects of ageing. The faeces experienced dehydration, mainly affecting moisture-dependent characteristics. Moisture content decreased from 79%wt to 26%wt, and water activity of 0.67, which corresponds mainly to the removal of interstitial bound water, reducing mass by 72%. The decreasing moisture content expectantly reduced drying ability, flowability and thermal properties (heat capacity and thermal conductivity). During this period, negligible biodegradation was recorded (volatile solids reduced by 3%), resulting in consistent chemical oxygen demand, particle size, carbon content and calorific values. Ammonium and nitrates decreased, but total nitrogen remained unchanged. Therefore, ageing affects nitrogen chemical forms and not nutrient composition. The findings demonstrate the benefits of source separation and in particular ventilated storage as a passive way to pre-treat and recover resources from faecal material.

## Highlights

• Faeces are dehydrated during their ageing, reducing in weight by 72% after 16 weeks.
• Ageing reduces water activity in faeces to 0.67 after 16 weeks of storage.
• Ageing mostly impacts moisture related properties, not energy and nutrient content.
• Ageing removes mainly interstitial bound water without causing any biodegradation.
• Ageing can improve the faeces drying performance and mechanical handling.

## Introduction

As of 2020, there were 3.6 billion people in the world without access to safe sanitation and 494 million still practice open defecation due to not having useable toilet facilities (WHO, 2021). Population growth statistics project a rise in the global generation of faeces from 7.0 × 10^11^ kg per annum in 2014 to 4.6 × 10^12^ kg per annum in 2030 (Berendes et al., 2018). An increase of this magnitude will significantly outpace sanitation service provision, especially in low-income areas already experiencing challenges in providing basic sanitation.

A potential sustainable solution is the separation and treatment of urine and faeces at the toilet user interface (source separation) (Larsen et al., 2021), which has been gaining interest globally since the 1990s. Source separation is advantageous over traditional toilets due to the isolation of a nutrient rich urine stream, while passively dehydrating faeces to reduce mass and therefore transportation costs (Strande and Brdjanovic, 2014). These systems thereby promote circular economy opportunity with a closed-loop nutrient cycle gaining attention in low, medium and high income countries alike (Freguia et al., 2021, Larsen et al., 2021). An established source separation system is the urine diversion dry toilet (UDDT), which diverts urine to percolate into the ground or a storage tank for reuse or treatment. Faeces are deposited and confined in a vault (Strande and Brdjanovic, 2014). In this scenario, faeces undergo physicochemical changes while stored in-situ for an extended period (WHO, 2019). More recently, the launch of the Reinvent The Toilet Challenge (RTTC) in 2011, initiated the development of innovative non-sewered sanitation technologies, funded by the Bill & Melinda Gates Foundation (BMGF, 2012). Within these systems, the solid and liquid streams are separated at the user interface (front-end), then safely treated in different modules on the back-end.

Conventional UDDTs and innovative sanitation technologies with source separation store the faecal material for a few days to a few years, depending on the type of system. During storage, the properties of faeces can undergo modifications in-situ, which can impact the pathogen, nutrient and organic content in the faeces (Strande and Brdjanovic, 2014). A substantial amount of data is available on the initial properties of fresh faeces (Almeida et al., 1999, Zavala et al., 2002, Nwaneri et al., 2008, Onabanjo et al., 2016a, Woolley et al., 2014). However, there is limited understanding and data on how the characteristics of fresh human faeces change from the time of generation. Understanding the physiochemical modifications of fresh faeces during ageing under in-situ containment is critical information for designing dry sanitation systems, treatment, and disposal/resource recovery strategies.

This study aims to temporally evaluate the properties of human faeces from the time of generation to 16 weeks of storage, replicating dry onsite sanitation systems that have ventilation and employ source separation in their design. The specific objectives are to determine the impact of faecal ageing on the material properties, downstream treatability and possible reuse pathways. The focus of this work was on the change in properties of fresh faeces over time in storage, so urine, anal cleansing material and flush water were excluded. The outcome from this investigation can be applied in conventional UDDTs or other sanitation technologies where the faeces are stored for a given period prior to their treatment or disposal.

## Materials and methods

### Fresh faeces sampling

The faecal samples for this study were obtained from donors from Howard College campus at the University of KwaZulu-Natal (UKZN), Durban, South Africa, and other areas in the college vicinity. An economic incentive was offered to volunteers to donate fresh faeces for the study. They were required to be over 18 years of age and willing to co-operate on the condition of anonymity. Donors received 1 L buckets (pre-weighed and numbered) to facilitate easier collection of faecal samples from the individual donors. The stipulation was to bring the stool in the bucket (no urine or anal cleansing material) to the laboratory within 24 hours. The study received a total of 73 samples from donors where each sample was immediately weighed, sealed, and placed in the cold room at 4 °C until use, to prevent microbial activity and preserve their physicochemical properties. Samples stayed under preservation until enough samples had been collected to cater to all experimental work. Therefore, the cold room storage time ranged from less than a day to one week depending on when the sample was brought in. The Biomedical Research Ethics Committee (BREC) from UKZN approved this study through the ethical clearance with reference number BREC/00000524/2019 before engaging potential volunteers and procuring samples.

### Experimental setup

**Fig. 1:**
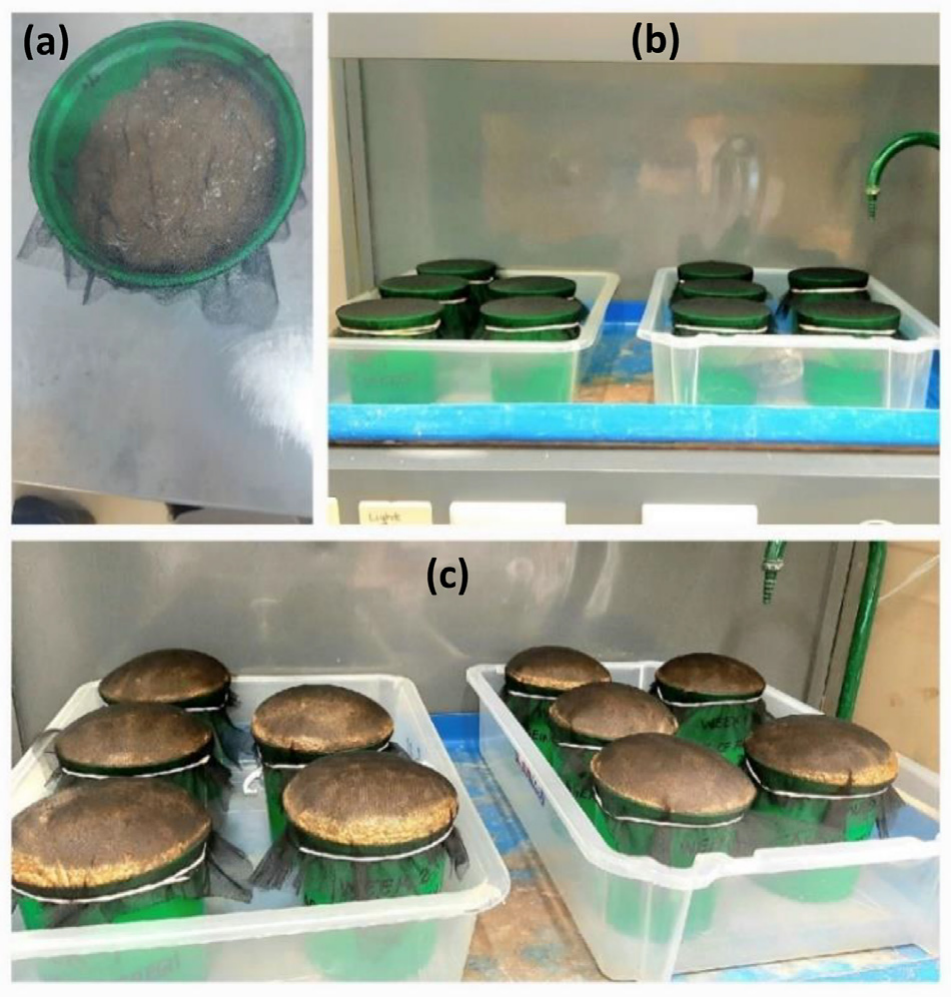
(a) 1 L bucket containing 900 g of fresh faeces - (b) buckets of fresh faeces in fume-hood (start of experiment) - (c) sample swelling after 24 hour-storage.

At the end of the collection period, all 73 samples were gently mixed using a polypropylene stick until a uniform colour was achieved, to resemble a homogenous composite. This mixing method was chosen to avoid altering structural properties such as the rheological properties. The composite was aliquoted into eleven 1 L buckets with an exposed surface area (111 cm^2^) to volume (1133 cm^3^) ratio of 1:11 (bottom diameter of 11 cm, an opening of 11.9 cm and a height of 11 cm). Each bucket contained 900 g of fresh faeces (Fig. 1a).

One aliquot was labelled Day 0 and was analysed on the day of mixing to establish the initial properties of fresh faeces before ageing. The remaining buckets were labelled Week 1 to Week 8, Week 12 and Week 16. The bucket openings were covered with mesh fabric to keep insects from accessing the faeces while allowing moisture and odours to escape. The containers were kept open to mimic ventilated conditions in onsite sanitation facilities. The ten aliquots were all ventilated in a fume hood at the minimum flowrate of 0.67 m/s, and left to age under ambient conditions for the number of weeks corresponding to their labels (Fig. 1a). After ageing over the desired timespan, the material in the bucket was gently homogenised through hand-mixing with a stainless-steel rod before analyses. Aged material with crusty surfaces had their crusts broken down first using the rod before mixing all the bucket contents.

There were unforeseen qualitative changes in the first week of storage. Therefore, it was decided to collect a second batch of fresh faeces and study the changes during the initial week of storage in more detail under similar conditions. Similar results were obtained between the two experiment batches, and therefore, only results from the first batch (with the exception of rheological measurements) will be presented in this paper.

### Characterisation of samples

The analyses of the samples were performed according to the Methods for Faecal Sludge Analysis (Velkushanova et al., 2021).

#### Moisture content and volatile solids analysis

Samples of mass 10 – 20 g were dried in the oven at 105 °C for 24 hours to determine moisture content and volatile solids (VS) by igniting the dried residue from the moisture content analysis in a muffle furnace at 550 °C for 2 hours.

#### Drying curves

The influence of ageing on the drying behaviour of faecal material was determined from drying tests. A 1.0 – 1.5 g thin film of homogenised faeces spread on a 90 mm diameter aluminium tray was completely dried at 105 °C using a *Radwag Max 50* Thermal Moisture Analyser. The balance in the analyser continuously weighed the sample during drying, and the sample weight was captured every 30 seconds, which allowed determination of the kinetics of the process, i.e. the drying curve (moisture content versus time) and the Krischer curve (drying rate versus moisture content). The Moisture Analyser directly computed the drying curve, whereas the Krischer curve was obtained by the user by calculating the drying rate (= mass differential / time differential) at different instants. The drying rate was normalised to g/min moisture removed assuming all samples had the same surface area (6361 mm^2^).

#### Water activity

Water activity estimates the degree to which moisture is bound to the solids in the faeces. An *AquaLab Tunable Diode Laser-TDL* water activity meter measured the water activity of a 10 ml sample at 25 °C. The instrument maintained this temperature at an accuracy of ±0.005 °C.

#### Rheology

Viscosity and shear stress tests on ageing faecal matter were performed at 25 °C using an *Anton Paar Rheometer MCR72*. The flow and deformation of samples were investigated using rotational tests at controlled shear rates. Each test subjected 100 – 150 g of faecal samples to a shear rate that increased from 0.1 to 1 000 s^−1^ for approximately 15 minutes, using a vane in cup geometry. The *Rheocompas*s^TM^ software captured the rheology data, shear strain-shear rate and viscosity-shear rate relationships on a logarithmic scale.

Yield stress is the lowest shear stress magnitude that needs to be applied to faeces to initiate their flow. The property was determined by plotting viscosity against shear stress (Septien et al., 2018). The yield stress is the shear stress value where faeces transition from elastic to viscous deformation. This point is characterised by a change of slope in the graph when the viscosity starts to drop. The smooth decline in viscosity with increasing shear stress before the yield stress corresponds to the elastic deformation.

#### Particle size analysis

The change in particle size of the faecal matter samples at different storage times was analysed using the *Malvern Mastersizer 3000* particle size analyser. The laser diffraction method used by this analyser assumes particles as spherical. Sample preparation involved dispersing a quarter of a 4 ml-spatula of faecal material in enough water to form a well-mixed slurry in a crucible. Slurry droplets were dispersed in 600 ml of water, and the instrument measured the particle size.

#### Chemical analysis

The chemical oxygen demand (COD), ammonium (NH_4_^+^), nitrate (NO_3_^−^), carbon (C) and nitrogen (N) content of samples were analysed to establish how the chemical composition of fresh faeces evolved whilst undergoing ageing. COD, NH_4_^+^ and NO_3_^−^ were measured using spectrophotometry (Spectroquant® cell tests, Merck), while C and N were measured using a *LECO TruMac* CNS analyser using pre-weighed masses of 0.1 – 0.2 g of faeces.

#### Calorific value

Faecal calorific values were measured by fully combusting 0.5 – 0.7 g of dried material in pure oxygen using a *Parr 6200 Calorimeter™*. The heat released by the material after oxidation determined the calorific value. Samples were oven-dried at 105 °C and ground into pellet/powder form prior to testing the calorific content with the results presented on a dry basis.

#### Thermal properties

The thermal conductivity and heat capacity were measured using a *C-Therm TCI™* thermal conductivity analyser. A 1.88 ml portion from the homogenised aliquot sample of known density was placed covering the sensor. The thermal properties were determined using the *C-Therm TCi* software based on the sensor's response to the heat applied.

### Statistical analysis

All tests were conducted in triplicates to ensure replicability; however, some rheology tests were duplicated due to insufficient sample mass. The standard deviation with respect to the average parameter value was calculated using Microsoft Excel to characterise the measurement uncertainty. The error bars in the figures represent the standard deviation of a triplicated experiment. Regression analysis established goodness of fit between different parameters for some properties.

## Results and discussion

### Qualitative observations

Within 24 hours of placing the samples in the fume cupboard, an interesting observation was that the fresh faeces began to swell (Fig. 1). The 1 L buckets could not contain the increasing sample volume (Fig. 1c), and subsequently, the samples were transferred to 2 L containers (with a bottom diameter of 14.5 cm, an opening of 16.5 cm and a height of 12.5 cm, exposed surface area to volume ratio of 1:11), where swelling continued for the following three days. During the transfer of the faecal material from the 1 L to the 2 L bucket, the mesh fabric in contact with the sample surface had noticeably fused with the surface layer of fresh faeces. After four days, the swelling stopped, and the volume began shrinking from day five and continued to reduce as the faecal material aged. The volume was not measured due to the shape of the samples, however there was a change in mass from day 0 to week 16 which was calculated to reduce by ∼72%.

The swelling observed in fresh faeces could be due to dissolved gasses which also cause ‘floaters’ (Aalam et al. (2022). Floaters are caused by the metabolism of carbohydrates by gasogenic gut microbiota (e.g. *Bacteroides ovatus*), producing gases such as H_2_ and CO_2_, which are subsequently converted to CH_4_ and H_2_S by hydrogenotrophic bacteria (Aalam et al., 2022). Different types of faecal samples were received and mixed in the study, and it is assumed they included floaters. Therefore, the sample swelling in this study was possibly caused by the trapped gases escaping from the faecal matter and homogenisation of the faecal samples into a composite mixture may have further aided the gas release. The sample swelling is not expected to affect processes occurring during storage or downstream processes in real sanitation systems since it was a temporary phase lasting only four days in this study.

**Fig. 2:**
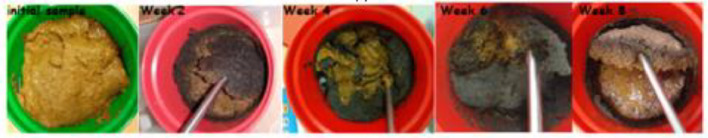
Progression of crust formation and volume shrinkage over 8 weeks.

Crust formed on the sample surface thickened with age (Fig. 2). The sample dried from the exterior surface to the interior, as noted from the dry crusty surface and moist core. Limited internal moisture transfer possibly caused this as if internal moisture is not transported fast enough to the material surface, superficial desiccation occurs at the surface (Léonard et al., 2004). The surface area to volume ratio is a key factor in faecal matter drying as it determines how much of the sample was exposed to the external environment to facilitate the drying process. The results in this study apply to a surface area to volume ratio of 1:11.

### Drying characteristics

**Fig. 3:**
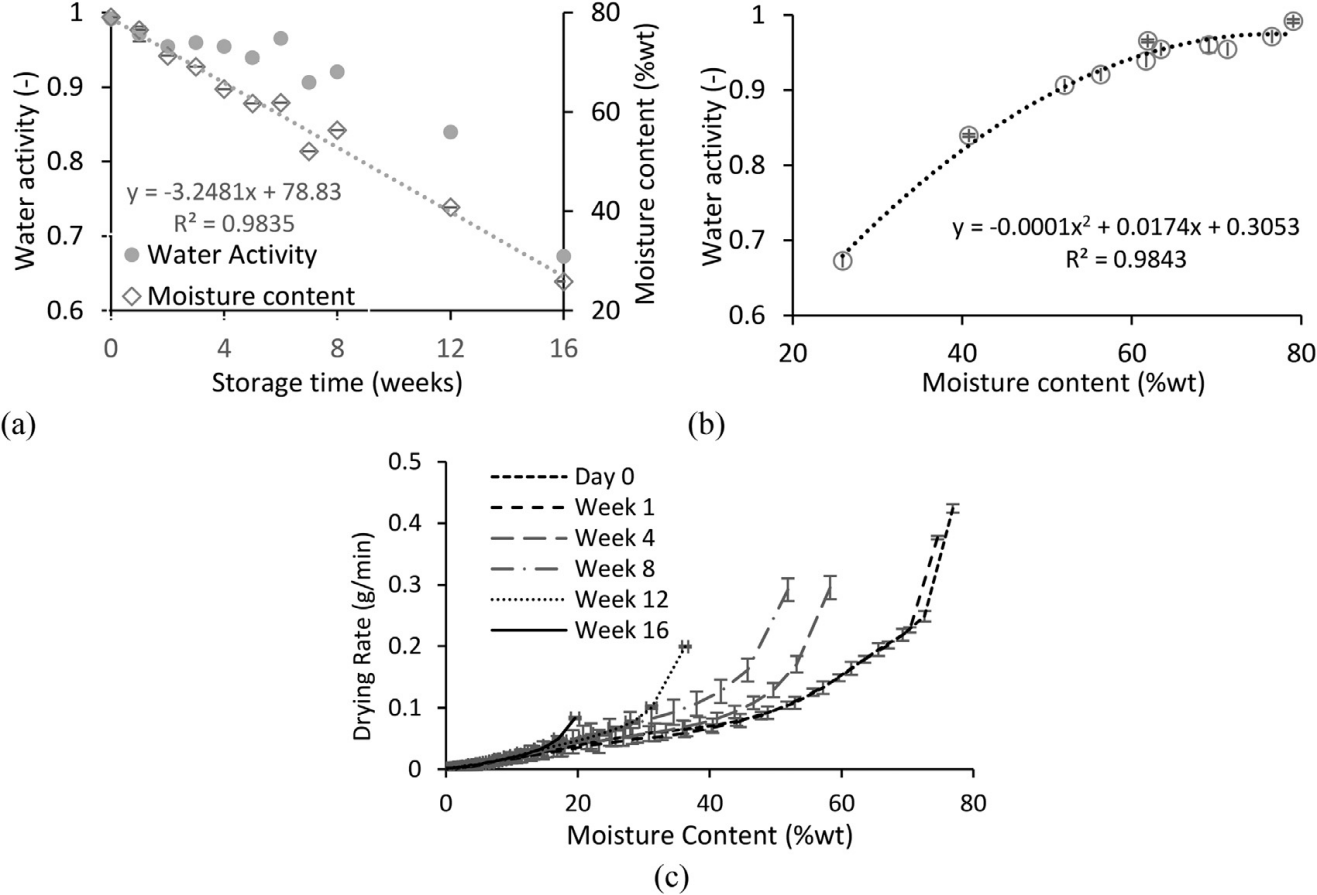
Evolution of drying characteristics of faeces over sixteen weeks in storage - (a) Moisture content and water activity as a function of storage time - (b) Water activity as a function of moisture content - (c) Instantaneous drying rate of faeces at different storage times. Error bars represent standard deviation of a triplicated experiment.

Sample moisture content measurements evaluated the dehydration effect of on-site containment (Fig. 3a and b), while water activity measurements estimated the fractions of unbound and bound moisture removed during the containment period (Fig. 3a and b). Drying curves were plotted to examine the drying ability of the faeces as a function of ageing, crucial in designing subsequent drying treatment processes (Fig. 3c).

#### Moisture content and water activity

The initial moisture content of fresh faeces was 79 %wt, which agreed with the 77 – 82 %wt range reported in literature (Almeida et al., 1999, Zavala et al., 2002, Nwaneri et al., 2008, Woolley et al., 2014, Remington, 2019). This corresponded with a water activity of 0.99 which was similarly reported by Remington (2019) of 0.93-1 (63-86 %wt). Over 16 weeks, moisture reduced from 79 to 26 %wt and water activity from 0.99 to 0.67 (Fig. 3a). Moisture content decreased with storage time. The water activity of the raw faeces was close to 1, which is consistent with the presence of unbound moisture in the samples. After week 1, the water activity begins to fall, suggesting that the remaining moisture is bound within the solids matrix (Fig. 3a and b). The parabolic profile of the water activity decrease looks like the shape corresponding to the interstitial moisture region in a typical sorption isotherm from sewage sludge (Vaxelaire and Cézac, 2004). These results indicate that faecal material gradually dried over time during storage losing mainly interstitial moisture, after an initial removal of unbound moisture. The trend in the current study was supported by other studies where faecal waste stored in conventional onsite toilets dehydrated over time (Zuma, 2016, Bakare, 2014, Wood and Buckley, 2013, Remington 2019, Getahun et al., 2020).

The implications from the reduction of moisture content and water activity could provide significant benefits for subsequent treatment. Firstly, the sticky phase which is one of the leading causes of sludge dryer failure from fouling and characterised between a MC of 50 to 70%wt for faeces (Mercer et al., 2021), is bypassed after 12 weeks of storage (MC < 41%wt). Secondly, studies on pathogenic faecal bacteria such as E. coli, salmonella and vibrio cholerae reported inactivation between a water activity of 0.62 - 0.91 (Mujumdar and Devahastin, 2000). However, these studies were investigated on a food substrate and therefore further research is required to examine water activity related pathogen inactivation in faeces. In a study where Ascaris eggs were stored in faecal sludge from on-site sanitation during a long period (Naidoo et al. 2020), it was observed that the Ascaris eggs development viability drastically decreased after four weeks of storage for moisture content below 30%, corresponding to a water activity of approximately 0.91 according to Getahun et al. (2020) using the same type of sludge. This result suggests that Ascaris eggs can also be deactivated below a water activity of 0.91.

#### Drying kinetics

Fig. 3c presents the drying curves of the samples after ageing expressed as drying rate at reducing moisture content. All samples had the same surface area for drying and are compared based upon drying rate in g/min moisture removed. As observed in Fig. 3c, the drying rate dropped quickly for the samples at different storage times, suggesting an increasing mass transfer limitation of the moisture inside the material to the surface as drying proceeded. A trend in the drying curves as a function of the sample age was noted where initial drying rates were lower as the samples aged. For example, the initial drying rate was of 0.4 g/min for the raw faecal sample (day 0) whereas the sample after 8 weeks of storage exhibited a drying rate of approximately 0.3 g/min.

Interestingly, it could be noted that a more aged sample exhibited starting higher drying rates than a less aged sample (with a higher moisture content) after achieving the same level of moisture content. For example, the faecal sample at day 0 shown a drying rate of approximately 0.1 g/min at 50%wt moisture content whereas the sample with an age of 8 weeks presented a drying rate of approximately 0.3 g/min at the same moisture content. The ageing process seemed to have allowed moisture redistribution within the samples during the natural drying process. This behaviour is analogous to intermittent drying mechanism wherein materials are rested in between drying periods to allow moisture redistribution within the sample (Barbosa de Lima et al., 2016), leading to higher drying rates when the process was resumed. Eventually, the drying rates from all the samples at different ages converged into the same pattern, implying that drying occurred in the same way at the last phase regardless the age of the samples.

From the results obtained in this section, it is evident that aging passively improves the treatability of the sludge for drying processes, particularly at the first phase of the transformation.

### Mechanical properties

**Fig. 4:**
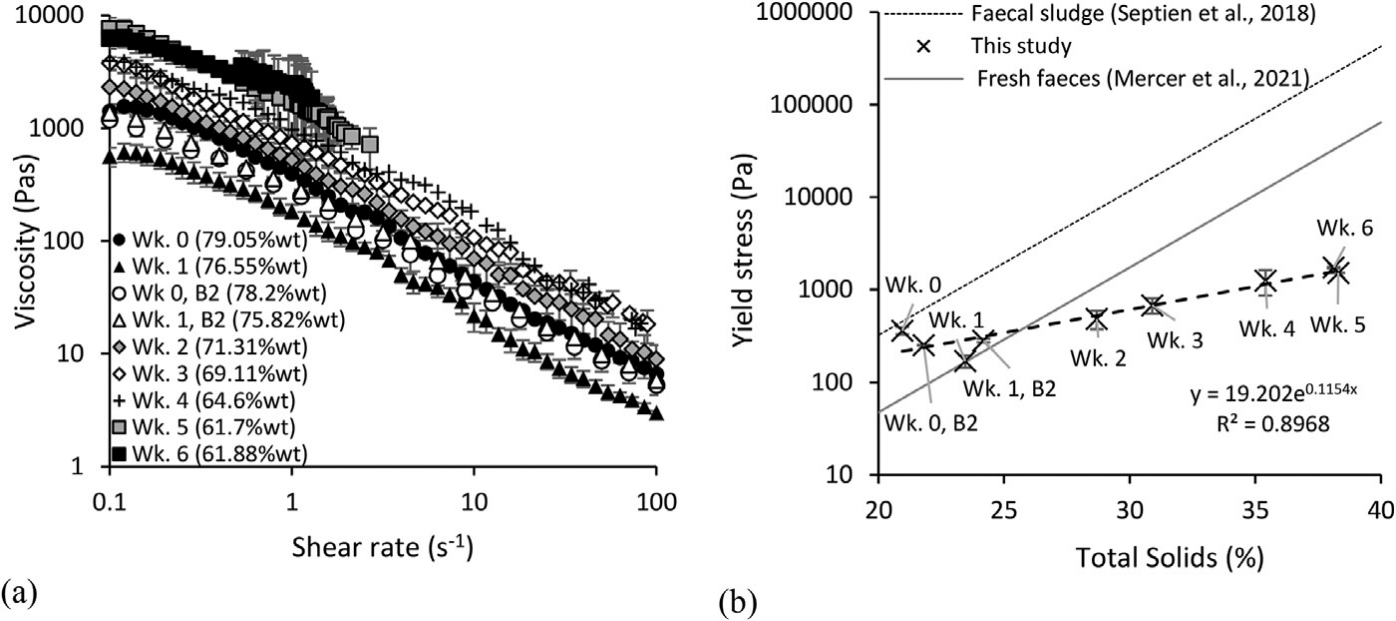
Rheological properties of faecal samples at different ages *-* (a) Viscosity against shear rate - (b) Yield stress against solids concentration. Note: week 0 and week 1 batch 2 (B2) viscosity and yield stress measurements were also included to demonstrate the impact of swelling as different degrees of swelling were experienced between batches which could have impacted rheological measurements . Error bars represent the standard deviation of a duplicated experiment.

**Fig. 5:**
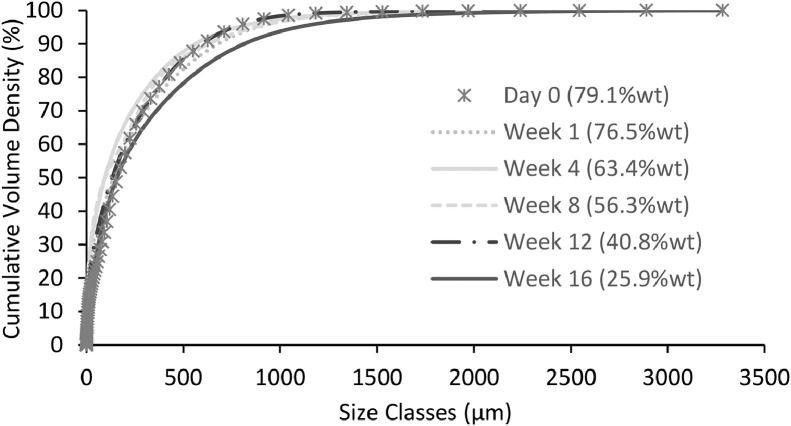
Cumulative particle size distribution of particles in faeces ageing over 16 weeks.

The mechanical properties of the faecal samples were measured to determine how sample fluidity evolved during ageing. Three parameters were investigated: viscosity as a function of shear rate (flow curves), yield stress and particle size distribution. The results are displayed in Fig. 4, Fig. 5.

#### Flow curves

Sample viscosity reduced with increasing shear rate, demonstrating shear-thinning properties (Fig. 4a), as also observed by Woolley et al. (2014) and Mercer et al. (2021) for fresh faeces. Generally, the viscosity increased with ageing due to the increased solids concentration from dehydration (Woolley et al., 2014, Septien et al., 2018). Removing moisture from the structural matrix of faeces limits particle and floc movement resulting in reduced flowability and increased viscosity. However, this was not the case for week 0 and week 1 of batch 1 as it was for batch 2. This could be a result of the greater degree of swelling observed in batch 1 compared to batch 2 after 5 days which could have decreased its viscosity to less than that of week 0. Beyond week 4, measurements were only possible at low shear rates due to increased viscosity as faecal matter aged, making it more difficult for the material to flow. There were no results from week 7 onwards because the samples were too dry to flow. Septien et al. (2018) and Mercer et al. (2021) noted that it was not possible to induce flow in faecal sludge and fresh faeces below a moisture content of 60%wt.

#### Yield stress

Yield stress increased exponentially from 144 to 1736 Pa during a storage time of 6 weeks and a corresponding solids concentration of 21 to 38%wt total solids (Fig. 4b). This exponential relationship is similarly observed by Septien et al. (2018) for partially digested pit latrine sludge (ash content ∼30%) and Mercer et al. (2021) for fresh faeces. However, it is interesting to note that the gradient of the line (Fig. 4b) is shallower for aging faeces than for fresh faeces and faecal sludge. Mercer et al. (2021) compared fresh faeces yield stress to non-sewered faecal sludge, and sewered wastewater sludges at identical solids concentrations. They observed that fresh faeces have a lower yield stress than faecal sludge and centralised wastewater sludge, and hypothesised that microbial assisted degradation in addition to the high shear history encountered by centralised wastewater sludges, facilitates particle size reduction and cell lysis leading to the of release extrapolymeric substances. These rigid flocs structures are known to require high compressive and shear stresses to initiate dewatering and flow.

The fact that increased storage promoted comparatively lower yield stresses compared to faecal sludge and other faeces matrices at the same total solid content implies that this method increased the treatability of faecal material by requiring lower energy for its mechanical handling. The mechanisms causing this phenomenon are unknown and requires further investigation.

#### Particle size distribution

Particle sizes for all samples ranged between 0.49 and 624 μm (Fig. 5). There was no observable trend as a function of the storage time with the particle size range remaining consistent during ageing as a similar mean particle diameter of 125−171 μm and median size of 92−153 μm was recorded. This lack of change could be because the samples were undisturbed and did not break down from shear, in addition to the minimal degradation (as discussed in the next section), known to reduce particle size. This is advantageous as smaller particle sizes will inhibit settling processes.

### Physicochemical properties

The physiochemical properties of the faeces at different storage times were determined to better understand the changes the material underwent as it aged and to evaluate the potential reuse of the aged faeces as fertilizer and biofuel through the analysis of organic, nitrogenous, and calorific content (Table 1).

**Table 1:** Physicochemical properties of the faecal samples after ageing over 16 weeks (mean±stdev).

Storage time (weeks)	Volatile solids (g/g dry sample)	C (g/g dry sample)	N (g/g dry sample)	COD (g/g dry sample)	NH_4_^+^ (g/g dry sample)	NO_3_^−^ (g/g dry sample)	Calorific value (MJ/kg dry sample)
0	0.856±0.001	0.51±0.06	0.06±0.01	1.02±0.03	0.031±0.0027	0.014±0.0011	20.91±0.14
1	0.842±0.006	0.46±0.00	0.06±0.01	0.67±0.02	0.012±0.0005	0.004±0.0010	21.31±0.16
2	0.847±0.001	0.50±0.01	0.07±0.01	1.46±0.11	0.013±0.0004	0.026±0.0030	21.55±0.17
3	0.842±0.005	0.50±0.04	0.07±0.00	1.25±0.03	0.018±0.0017	0.003±0.0007	21.22±0.26
4	0.845±0.004	0.48±0.04	0.07±0.00	1.57±0.09	0.013±0.0005	0.008±0.0003	21.50±0.06
5	0.838±0.004	0.49±0.02	0.06±0.00	1.40±0.19	0.014±0.0004	0.003±0.0007	21.56±0.09
6	0.844±0.001	0.51±0.02	0.07±0.00	1.52±0.09	0.014±0.0008	0.003±0.0007	21.77±0.11
7	0.836±0.002	0.48±0.01	0.06±0.00	1.35±0.05	0.014±0.0000	0.004±0.0002	21.03±0.0.16
8	0.836±0.005	0.40±0.00	0.05±0.00	1.30±0.05	0.014±0.0003	0.003±0.0003	22.33±0.38
12	0.837±0.000	0.53±0.02	0.08±0.02	1.33±0.11	0.013±0.0004	0.003±0.0003	21.83±0.05
16	0.827±0.004	0.49±0.02	0.07±0.02	1.15±0.01	0.009±0.0000	0.003±0.0005	21.60±0.34

#### Organic matter content

Initially, the volatile solids content was ∼0.86 g/g dry sample, close to 0.84 g/g dry sample measured for fresh faeces by Bakare (2014). This slightly decreased to 0.83 g/g dry sample, demonstrating minimal biodegradation.

Fresh faeces exhibited a COD of 1.02±0.03 g/g dry sample, which fell within the range of 0.567−1.45 g/g dry sample found in different studies on fresh faeces (Almeida et al., 1999, Zavala et al., 2002, Nwaneri et al., 2008, Rose et al., 2015, Remington, 2019). The disparity in fresh faeces' COD among studies could be caused by diet diversity in study participants, as Tilley (2014) and Zavala et al. (2002) suggested. No significant trend was discernible in how COD varied with increasing sample age, so the COD could be considered unimpacted by ageing. The COD behaviour in this study was different from trends found in studies on faecal sludge from dry pit toilets, where it declined during storage. However, pit sludge is contained onsite for years which could be long enough for the COD to decrease (Nwaneri et al., 2008, Bakare, 2014, Zuma et al., 2015).

Carbon was the primary element in the faecal samples with a content of 0.5 g/g dry sample, representing half the mass of the material, as also found by Onabanjo et al. (2016b). According to Harder et al. (2019) and Muspratt et al. (2014), carbon content can reduce with time when biodegradable carbon breaks down. However, the faecal carbon appeared unvaried with increasing sample age.

In summary, minimal degradation was recorded, as the method of ventilated dry storage may slow both anaerobic and aerobic degradation processes, which are usually encountered in non-source separated faecal sludge and centralised sewage sludge respectively. This result could be because material dehydration reduced the microbial activity responsible for degradation, through decreasing water activity.

#### Nitrogenous compounds content

Nitrogen is crucial in the metabolism and growth of plants and usually gets assimilated as ammonium and nitrate. Therefore, it is essential to know the content of nitrogen and nitrogenous molecular nutrients in faeces while ageing to determine their value as an agricultural product. The total nitrogen remained in the range of 0.05-0.08 g/g dry sample, representing 5-8% of the faeces on a dry composition, similar to study findings by Feachem and Cairncross (1978). However, there was a difference in ammonium and nitrate content between the fresh and aged faeces. Faecal samples initially contained 0.031 g/g dry sample of ammonium and 0.009 g/g dry sample of nitrates and after 16 weeks of storage 0.014 g/g dry sample and 0.003 g/g dry sample respectively.

When Septien et al. (2020) studied the impact of drying on the physicochemical properties of faecal sludge, ammonium, nitrites, and nitrates decreased as drying proceeded, but the total nitrogen content remained constant. The authors suggested that the decrease in the nitrogenous molecular compounds content reflected changes in the chemical form of nitrogen. They hypothesised that nitrogen bound with the dry bone structure of the faecal material, thus decreasing its chemical form as individual molecules. This explanation could also be applied to faeces during its aging.

#### Calorific value

The average calorific value for fresh faeces in this study was ∼ 22 MJ/kg dry sample, lower than that measured by Onabanjo et al. (2016a), namely 25 MJ/kg dry sample. However, it was on the upper limit of the range reported by Muspratt et al. (2014), i.e., 8.0 -23.0 MJ/kg dry sample, for conventional biosolids and faecal sludge. Importantly, this value remained stable over 16 weeks of storage, due to minimal degradation (stable volatile solids content). Sample drying occurred under ambient conditions (∼25 °C) but it did not impact the net calorific value. According to Getahun et al. (2020), the net calorific value of faecal sludge is not affected by drying at temperatures below 150 °C, but temperatures higher than 200 °C can cause a decrease. These results demonstrate how a passive drying process, which requires no external heat energy, enables full biofuel energy potential.

### Thermal properties

The thermal properties, including the thermal conductivity and heat capacity, were measured for the faecal samples for 16 weeks. The thermal conductivity determines the inherent ability of the samples to conduct heat. The heat capacity indicates the amount of heat required to increase the temperature of the faecal samples.

#### Thermal conductivity

**Fig. 6:**
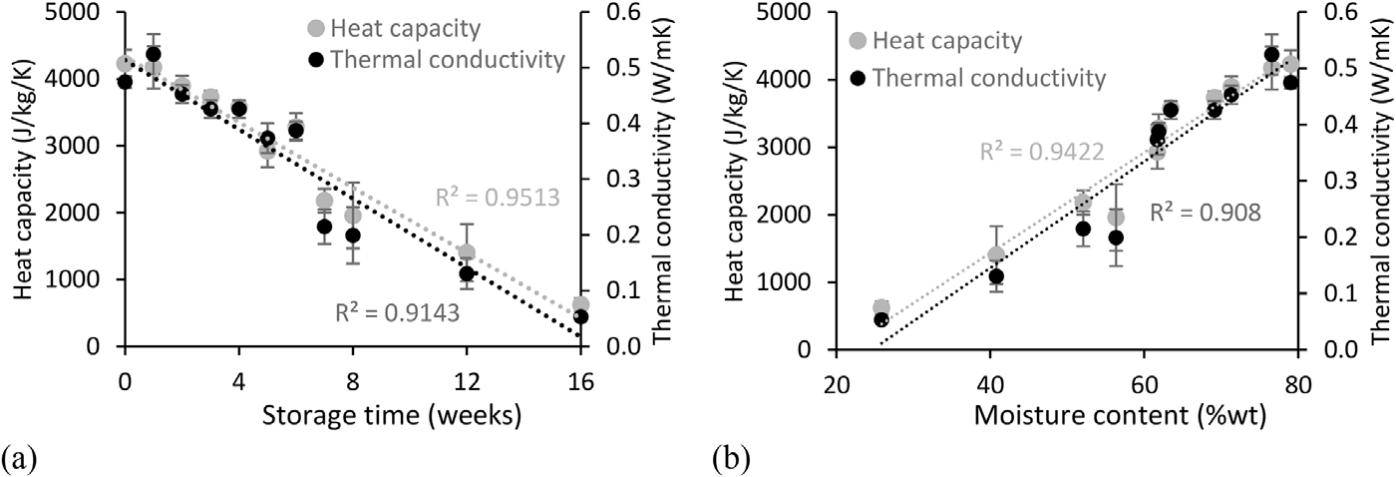
Thermal properties of faeces over 16 weeks - (a) Thermal conductivity and heat capacity as a function of storage time - (b) Thermal conductivity and heat capacity as a function of the moisture content. Error bars represent standard deviation of a triplicated experiment.

Thermal conductivity varied during storage by decreasing over time (Fig. 6a). The initial value of fresh faeces of around 0.5 W/m/K was close to that of pure water (0.6 W/m/K) due to the high moisture content of the material (79%wt). Thermal conductivity subsequently decreased during storage, linked to faecal matter drying as the main contributor to thermal conductivity is water (Fig. 6b). Septien et al. (2020) found a similar trend while drying faecal sludge. A lower thermal conductivity is a negative aspect for thermal treatment or reuse of faecal matter as biofuel as the heating of the material would take more time. However, this could be a positive aspect if the faeces are to be reused as insulating material (for example as a construction material).

#### Heat capacity

Similar to the thermal conductivity, the heat capacity experienced a reduction over time, also related to the loss in moisture from the samples during storage (Fig. 6a and b). Heat capacity had a starting value of 4 230 J/kg/K, close to that of pure water (∼4 180 J/kg/K). As the moisture content decreased to 29%wt after 16 weeks of storage, the heat capacity reduced to 620 J/kg/K, comparable to the value for air at 700 J/kg/K. A lower heat capacity is positive for the reuse of the aged faeces as biofuel or further treatment in a thermal process, as it would require less thermal energy for its heating.

## Outlook

This study has evidenced that ventilated faecal storage passively removes water from faeces without compromising the agricultural or biofuel resource recovery potential. The implications for sustainable non-sewered sanitation are substantial. Firstly, the heat energy saved to reduce 900 g of faeces from a moisture content of 79.05%wt to 25.9%wt is at least 1500 kJ, calculated from the latent heat of pure water vaporisation and without considering the increased energy required for removing bound water at lower moisture contents. This removal of moisture resulted in a mass reduction of 72%, which drastically reduces transport costs. The extent of drying after 16 weeks provided sufficient pre-treatment for the combustion of faeces as autothermal combustion conditions were met (MC <30%wt, Onabanjo et al., 2016a), which would further treat the material to a friable, safe to handle product, while recovering calorific energy which also presents an option for direct household reuse. This reduction of moisture also bypassed the sticky phase of faeces (50-70%wt, Mercer et al., 2021), which mitigates fouling in downstream solids treatment processes.

Secondly, the slow drying method improved the treatability of the material. We hypothesise that it allowed for moisture distribution within the faecal matter, analogous to intermittent drying (Barbosa de Lima et al., 2016). This promoted faster drying rates of older faeces at comparable moisture contents to fresher faeces. This method also impacted the rheology of faeces with the aged faeces possessing a lower yield stress than fresh faeces at the same solids concentration.

This investigation informs how the physicochemical properties of a volume of faeces temporally evolves in a static system. In a dynamic real-life system, such as that of current UDDTs, the continuous use of the toilet promotes the piling of faeces on top of each other, reducing the surface area to volume ratio and therefore drying rate. In the South African context, a double vault system is practiced whereby when one vault is full, faeces are collected in the other vault, while the original vault is ventilated and stored until collection (Strande and Brdjanovic, 2014). Subsequently, storage usually requires 1 year. The key design consideration for achieving the results from this study in practice are (a) the separation of fresher faeces from the older faeces, (b) equal or greater surface area to volume ratio (c) prevention of fly contact where larvae would change the material properties and aid as a pathogen vector. For example, a conveyance method could be put in place such as a screw conveyor (surrounded by a mesh for ventilation and fly prevention) which is occasionally rotated by a lever to move ageing faeces along the screw.

This research could also apply to innovative household scale toilets such as the Reinvented Toilets (BMGF, 2012). These systems typically source separate. In contrast to UDDTs, immediate solids processing occurs onsite, facilitated by energy intensive dewatering and drying pre-treatment. If the solids can be passively recovered and stored in a separate chamber (with a conveyance mechanism to separate fresher faeces on older ones), the vision of off-grid household-scale sanitation treatment and resource recovery could be realised.

## Conclusion

In this study, fresh faeces were stored under ambient conditions to simulate onsite dry toilets, and different properties were temporally measured to characterise faecal matter transformation by aging. Ageing reduced faeces in weight by 72% from a starting moisture content of 79 to 27%wt after 16 weeks of storage, which substantially reduces transport costs and treatment footprint. The dehydration of the faeces with storage modified the rheological and thermal properties, by increasing its viscosity and yield stress, and decreasing its thermal conductivity and heat capacity.

The water activity results indicated that unbound moisture was removed from faeces after 1 week, and interstitial moisture mostly removed after 16 weeks of storage, which coincided with overcoming the sticky phase of faeces. Not only does this study demonstrate that bound water can be removed passively, it has also evidenced that the treatability of the material improves, through greater drying rates and lower yield stress values at comparable moisture contents to fresher faeces.

Minimal degradation of the faecal material was observed, owing to the separation from a liquid matrix (such as urine and flush water) which encourages biochemical reactions, and ventilated conditions which prevent anaerobic digestion. This therefore differentiates the material to other aged faecal sludge types such as septic tank or pit latrine sludge. The faecal material was able to maintain a composition rich in organic matter, nitrogen and a high calorific content, promoting aged faecal material as an attractive alternative for crop-farming products and biofuel, and even construction material (thanks to its low thermal conductivity leading to interesting thermal insulation characteristics).

This study showed the benefits of faeces storage under ventilated conditions. The outcomes from this investigation demonstrated that faecal dry storage after source separation is a passive, yet effective method to dry and recover resources from faeces, which is relevant for the global demand for sanitation facilities. However, to enable this in practice, existing source separation systems must incorporate a design which separates fresher faeces from older ones. Further research is warranted to determine the impact of different surface area to volume ratios to understand how to design and optimise passive dehydration chambers, coupled with evaluating safe handling by pathogen inactivation. It would also be interesting to understand the specific mechanisms to why source separated aging improves material treatability in terms of drying rate and rheology and to determine whether it also applies to dewatering and stickiness.

## Funding

This research work was supported by the 10.13039/100000865Bill & Melinda Gates Foundation [Grant number OPP1069575].

## CRediT authorship contribution statement

**T.M. Chatema:** Conceptualization, Methodology, Validation, Formal analysis, Data curation, Writing – original draft, Investigation, Visualization. **E. Mercer:** Writing – review & editing. **S. Septien:** Conceptualization, Methodology, Writing – review & editing, Visualization, Supervision, Project administration. **J. Pocock:** Conceptualization, Methodology, Writing – review & editing, Supervision. **C.A. Buckley:** Conceptualization, Supervision, Project administration, Funding acquisition.

## Declaration of Competing Interests

The authors declare that they have no known competing financial interests or personal relationships that could have appeared to influence the work reported in this paper.

## Data Availability

Data will be made available on request.
